# Estimation of creatinine clearance using plasma creatinine or cystatin C: a secondary analysis of two pharmacokinetic studies in surgical ICU patients

**DOI:** 10.1186/s12871-015-0043-7

**Published:** 2015-04-28

**Authors:** Thomas Steinke, Stefan Moritz, Stefanie Beck, Carsten Gnewuch, Martin G Kees

**Affiliations:** 1 Department of Anaesthesiology and Surgical Intensive Care, University Hospital of Halle (Saale), Ernst-Grube-Str. 40, 06120 Halle (Saale), Germany; 2 Department of Anesthesiology, University Hospital Hamburg-Eppendorf, Martini-Str. 52, 20246 Hamburg, Germany; 3 Institute for Clinical Chemistry and Laboratory Medicine, Regensburg University Medical Center, Franz-Josef-Strauß-Allee 11, 93053 Regensburg, Germany; 4 Department of Anesthesiology and Intensive Care, Charité Universitätsmedizin Berlin ‐ Campus Benjamin Franklin, Hindenburgdamm 30, 12200 Berlin, Germany; 5 Department of Clinical Pharmacy and Biochemistry, Institute of Pharmacy, Freie Universitaet Berlin, Kelchstr. 31, 12169 Berlin, Germany

**Keywords:** Pharmacokinetics, Drug dosing, Glomerular filtration

## Abstract

**Background:**

In ICU patients, glomerular filtration is often impaired, but also supraphysiological values are observed (“augmented renal clearance”, >130 mL/min/1.73 m^2^). Renally eliminated drugs (e.g. many antibiotics) must be adjusted accordingly, which requires a quantitative measure of renal function throughout all the range of clinically encountered values. Estimation from plasma creatinine is standard, but cystatin C may be a valuable alternative.

**Methods:**

This was a secondary analysis of renal function parameters in 100 ICU patients from two pharmacokinetic studies on vancomycin and betalactam antibiotics. Estimated clearance values obtained by the Cockcroft-Gault formula (eCL_CG_), the CKD-EPI formula (eCL_CKD-EPI_) or the cystatin C based Hoek formula (eCL_Hoek_) were compared with the measured endogenous creatinine clearance (CL_CR_). Agreement of values was assessed by modified Bland-Altman plots and by calculating bias (median error) and precision (median absolute error). Sensitivity and specificity of estimates to identify patients with reduced (<60 mL/min/1.73 m^2^) or augmented (>130 mL/min/1.73 m^2^) CL_CR_ were calculated.

**Results:**

The CL_CR_ was well distributed from highly compromised to supraphysiological values (median 73.2, range 16.8-234 mL/min/1.73 m^2^), even when plasma creatinine was not elevated (≤0.8 mg/dL for women, ≤1.1 mg/dL for men). Bias and precision were +13.5 mL/min/1.73 m^2^ and ±18.5 mL/min/1.73 m^2^ for eCL_CG_, +7.59 and ±16.8 mL/min/1.73 m^2^ for eCL_CKD-EPI_, and -4.15 and ±12.9 mL/min/1.73 m^2^ for eCL_Hoek_, respectively, with eCL_Hoek_ being more precise than the other two (p < 0.05). The central 95% of observed errors fell between -59.8 and +250 mL/min/1.73 m^2^ for eCL_CG_, -83.9 and +79.8 mL/min/1.73 m^2^ for eCL_CKD-EPI_, and -103 and +27.9 mL/min/1.73 m^2^ for eCL_Hoek_. Augmented renal clearance was underestimated by eCL_CKD-EPI_ and eCL_Hoek_. Patients with reduced CL_CR_ were identified with good specificity by eCL_CG_, eCL_CKD-EPI_ and eCL_Hoek_ (0.95, 0.97 and 0.91, respectively), but with less sensitivity (0.55, 0.55 and 0.83). For augmented renal clearance, specificity was 0.81, 0.96 and 0.96, but sensitivity only 0.69, 0.25 and 0.38.

**Conclusions:**

Normal plasma creatinine concentrations can be highly misleading in ICU patients. Agreement of the cystatin C based eCL_Hoek_ with CL_CR_ is better than that of the creatinine based eCL_CG_ or eCL_CKD-EPI_. Detection and quantification of augmented renal clearance by estimates is problematic, and should rather rely on CL_CR_.

**Electronic supplementary material:**

The online version of this article (doi:10.1186/s12871-015-0043-7) contains supplementary material, which is available to authorized users.

## Background

In critical care, renal function receives much attention because deteriorations are frequently part of syndromes such as sepsis or low-output, and often associated with a dire prognosis [1]. Early recognition may offer the chance of successful treatment and hopefully prevention of progression to renal failure. For this purpose, the diagnostic approach must be able to discriminate normal from abnormal renal function, and to differentiate the latter into a limited number of degrees of severity. Despite all its drawbacks, plasma creatinine is still recommended as the primary biomarker for the evolution of acute kidney injury [1]. Since plasma creatinine (and other markers) can only start to rise when renal function is already compromised, research is rather directed towards identification and validation of markers of the preceding tissue damage, e.g. of cell cycle arrest [2,3].

A separate issue is the necessity to adjust the dose of drugs with predominantly renal elimination to renal function. Many antibiotics, e.g. aminoglycosides, glycopeptides, betalactams and part of the fluoroquinolones, fall into this category. Recommendations by the manufacturers usually suggest a similar approach, with one dosing regimen for patients with „normal“ renal function, and two or three adjusted dosing regimens for patients with reduced creatinine clearance, often starting in the range of <50-80 ml/min. It is clear that patients with „normal“ renal function will thus exhibit a high variability of actual antibiotic exposure.

In the ICU, three factors make this approach particularly problematic. First, infections are likely to be more severe and rather caused by less susceptible pathogens [4]. The risk of underdosing is therefore more substantial than in less severe cases. Second, some patients without signs of renal impairment will actually not just have a „normal“, but a supraphysiological creatinine clearance (>130 ml/min/1.73 m^2^). The term „augmented renal clearance“ has been coined for this phenomenon few years ago [5], and its prevalence in ICU patients without evidence of kidney injury was recently reported to be up to 65% in the first week of treatment [6]. Third, creatinine clearance is particularly difficult to estimate in ICU patients. Estimations based on plasma creatinine rely on the assumptions that the endogenous production of creatinine is stable and can be predicted reasonably well from patient characteristics (e.g. age, body weight and sex for the Cockcroft-Gault formula [7]). These assumptions are certainly questionable in ICU patients, and important discrepancies have been shown between the available estimation formulae [8-10]. Particularly, plasma creatinine concentrations within the normal range have been shown to reflect creatinine clearances both considerably below and above the normal range [6,11].

Cystatin C is an alternative endogenous marker, which is produced in a fairly constant rate by all nucleated cells and subject to glomerular filtration only [12]. Albeit promising and already implemented into clinical guidelines for chronic kidney disease [13], the value of the latter in ICU patients is still uncertain. In the present analysis, we compare the agreement of the estimated clearance of the Cockcroft-Gault formula, the CKD-EPI formula (both based on plasma creatinine), and the Hoek formula based on plasma cystatin C with the measured endogenous creatinine clearance [7,14,15], as well as their respective ability to detect patients with reduced or augmented endogenous creatinine clearance.

## Methods

### Study design

All data presented in this analysis were obtained during two pharmacokinetic studies on vancomycin (study 1 [16]) and piperacillin/tazobactam, meropenem or ceftazidime (study 2; partly published in abstract form [17], manuscript in preparation), respectively, which were conducted at the surgical ICU of the Charité Universitätsmedizin Berlin – Campus Benjamin Franklin (study 1 and 2) and two surgical ICUs of the Hospital of the University of Halle (study 2). The study protocols were approved by the competent ethics committees (Berlin: EA4/113/07, EA4/029/11; Halle: 2012-95). Written informed consent for study participation and publication of anonymised clinical data was obtained from the patient or a legal representative. Adult patients who were treated with one of the study drugs and were not on renal replacement therapy were eligible for enrolment. Besides that, no specific criteria of exclusion (e.g. diuresis or plasma creatinine concentration) were applied.

The investigations took place when clinical conditions with regard to hemodynamics and renal function (in terms of vasopressor doses, fluid balance and diuresis) were considered to be stable. Although no exact limits were defined, patients who required a specific clinical assessment (e.g. fluid challenge), further diagnostics or a change of therapy (e.g. starting or stopping a vasopressor or diuretic) were considered to be unstable.

In study 1 (25 patients), urine was collected for 18 hours. During urine collection, four plasma concentrations of creatinine (every 6 hours) and two plasma concentrations of cystatin C (at the beginning and the end) were obtained. The mean values were used for calculations. In study 2, the sampling period was chosen as convenient, and plasma creatinine and cystatin C were determined in one sample during or at the end of the urine collection interval.

### Assessment of renal function

Plasma and urine creatinine were determined by a standardised enzymatic assay on an ADVIA 1800 chemistry system (ECRE_2; Siemens Healthcare Diagnostics, Eschborn, Germany). Plasma cystatin C was determined by a particle-enhanced immunonephelometric assay on a BN II system (N CYSC; Siemens Healthcare Diagnostics, Eschborn, Germany).

Renal function parameters were calculated according to the following formulae as values normalised to body surface area (mL/min/1.73 m^2^):

- °Measured endogenous creatinine clearance (normalised to 1.73 m^2^ body surface area):
- °CL_CR_ = (urine volume × urine creatinine)/(collection time x plasma creatinine)/BSA × 1.73
- °According to Cockcroft-Gault [7] (normalised to 1.73 m^2^ body surface area):
- °eCL_CG_ = (140 – age x weight)/(72 x plasma creatinine)/BSA x 1.73 x 0.85 for women
- °According to the CKD-EPI formula [14]:
- °eCL_CKD-EPI_ = 141 × min(plasma creatinine/κ, 1)^α^ × max(plasma creatinine/κ, 1)^-1.209^ × 0.993^age^ × 1.018 (for women)
- °κ = 0.7 for women, 0.9 for men; α = -0.329 for women, -0.411 for men
- °According to the Hoek formula [15]:
- °eCL_Hoek_ = (80.35/plasma cystatin C – 4.32)
- °Body surface area [18]:
- °BSA = 0.007184 × height^0.725^ × weight^0.425^

Units: plasma and urine creatinine in mg/dL, plasma cystatin C in mg/L, age in years, (total body) weight in kg, BSA in m^2^, height in cm.

### Statistical analysis

Microsoft Excel for Mac 2011 (Microsoft Corporation, Redmond, WA) was used for calculation of renal function parameters, GraphPad Prism version 6 for MacOSX (GraphPad Software, La Jolla, CA) was used for statistical calculations. For each estimate of clearance eCL_X_, a modified Bland-Altman plot (eCL_X_ - CL_CR_ vs. CL_CR_) was generated. (Whereas in a classical Bland-Altman plot the x-axis shows the mean value of the compared methods, CL_CR_ was preferred here so that individual patients could be identified throughout the three plots by the identical x-value; statistical calculations are not affected by this modification). Bias was defined as the median error (eCL_X_ – CL_CR_), and precision as the median absolute error (|eCL_X_ – CL_CR_|). The paired Wilcoxon test was used to test for differences between the absolute errors of the three estimates. Sensitivity and specificity to detect CL_CR_-values of <60 or >130 mL/min/1.73 m^2^ were calculated for the identical thresholds (<60 or >130 mL/min/1.73 m^2^), and compared by Fisher’s exact test. Receiver-operator characteristic curves (ROC) were generated. All p-values provided are two-tailed.

## Results

**Figure 1:**
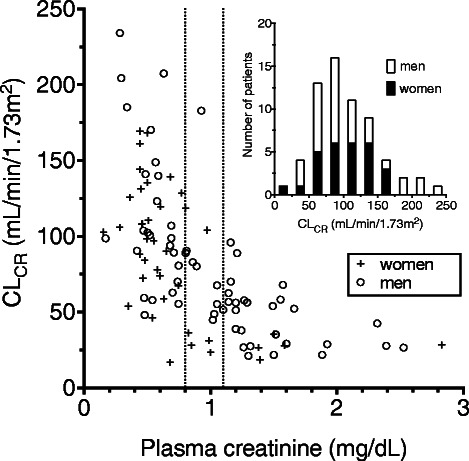
Measured creatinine clearance (CL_CR_) vs. plasma creatinine. Data were obtained from 100 ICU patients participating in pharmacokinetic studies on antibiotics. The inlet shows the distribution of the 63 patients with a plasma creatinine concentration below the upper reference limit (0.8 mg/dL for women, 1.1 mg/dL for men; vertical dotted lines).

In total, 100 patients contributed data to this analysis (25 from study 1 and 75 from study 2). Patient characteristics are summarised in Table 1, the full data set is available as Additional file 1. The majority of patients had undergone previous surgery (82 patients; mostly general surgery or neurosurgery), and were receiving the study antibiotics for intraabdominal infections (16 patients), nosocomial meningitis (11), nosocomial pneumonia (46) or other indications (27). Fourty patients received one or more vasopressors, and 61 were on mechanical ventilation on the day of the study. In study 1, in which 4 creatinine concentrations were determined during the 18 hours collection period, the median relative change between the first and the last value was ±4%. In one patient, it was -40% (a decline from 0.55 to 0.33 mg/dL), and less than ±18% in all others. In study 2, in which the duration of urine collection was not standardised, the median collection interval was 12 hours (range 1.92-22.75 hours), and at least 160 ml of urine were obtained. The patients’ renal function was spread over a wide range (CL_CR_ 16.8-234 mL/min/1.73 m^2^), particularly at plasma creatinine concentrations below the upper reference limit, i.e. 0.8 and 1.1 mg/dL for women and men, respectively (Figure 1). In 42 patients CL_CR_ was <60 mL/min/1.73 m^2^, and 16 patients presented augmented renal clearance (CL_CR_ > 130 mL/min/1.73 m^2^). In patients who had suffered a polytrauma, an isolated traumatic brain injury or a subarachnoid hemorrhage (conditions associated with augmented renal clearance) CL_CR_ was higher (median 102 mL/min/1.73 m^2^), and in 5 cases >130 mL/min/1.73 m^2^.

**Table 1:** **Patient characteristics (n = 100; 61 male, 39 female)**

	Median	Interquartile range	Range
Age (years)	66	57-74	21-85
Total body weight (kg)	78	70-90	42-125
Body height (cm)	170	165-175	138-190
BMI (kg/m^2^)	26.2	23.1-30.3	16.0-41.5
Length of hospitalisation (days)	16	10-27	2-95
Creatinine (mg/dL)	0.75	0.50-1.20	0.15-2.83
Cystatin C (mg/L)	1.12	0.76-1.73	0.50-4.21
CL_CR_ (mL/min/1.73 m^2^)	73.2	46.8-107	16.8-234
eCL_CG_ (mL/min/1.73 m^2^)	85.2	58.7-137	22.4-462
eCL_CKD-EPI_ (mL/min/1.73 m^2^)	87.2	59.4-108	16.7-196
eCL_Hoek_ (mL/min/1.73 m^2^)	67.3	42.2-101	14.8-156
APACHE II	18	13-23	5-33
SOFA	6	3-8	0-19

CL_CR_: measured endogenous creatinine clearance; eCL_CG_, eCL_CKD-EPI_, eCL_Hoek_: estimated clearance by the Cockcroft-Gault, CKD-EPI or Hoek formula; APACHE II: acute physiology and chronic health evaluation II score. SOFA: sequential organ failure assessment score.

**Figure 2:**
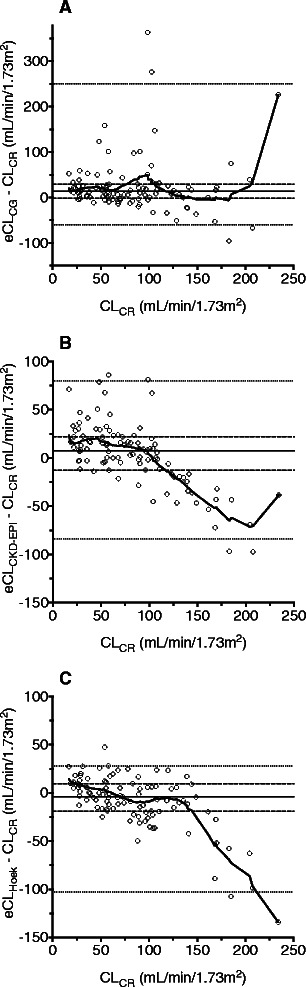
Agreement of estimated with measured creatinine clearance (CL_CR_) in 100 ICU patients. Estimation was done by **(A)** the Cockcroft-Gault formula (eCL_CG_), **(B)** the CKD-EPI formula (eCL_CKD-EPI_), or **(C)** the Hoek formula (eCL_Hoek_). Bold line: LOWESS curve. Horizontal solid line: median; dashed lines: 25th/75th percentile; dotted lines: 2.5th/97.5th percentile of observations. Note the larger scale of the y-axis in figure A.

The Bland-Altman plots are shown in Figure 2. Bias of eCL_CG_ was +13.5 mL/min/1.73 m^2^ and precision ±18.5 mL/min/1.73 m^2^, but excessive overestimations were frequent, with the central 95% of observed errors falling between -59.8 and +250 mL/min/1.73 m^2^. No trend was apparent throughout the range of observed values (Figure 2A). For eCL_CKD-EPI_, bias was +7.59 mL/min/1.73 m^2^ and precision ±16.8 mL/min/1.73 m^2^ (central 95%: -83.9 – +79.8 mL/min/1.73 m^2^). High values of CL_CR_ were underestimated (Figure 2B). The same was true for eCL_Hoek_, for which bias and precision were numerically lowest (-4.15 and ±12.9 mL/min/1.73 m^2^, respectively; central 95%: -103 – +27.9 mL/min/1.73 m^2^; Figure 2C). Absolute errors of eCL_CG_ and eCL_CKD-EPI_ were not statistically different, but both were different from that of eCL_Hoek_ (p < 0.05).

**Figure 3:**
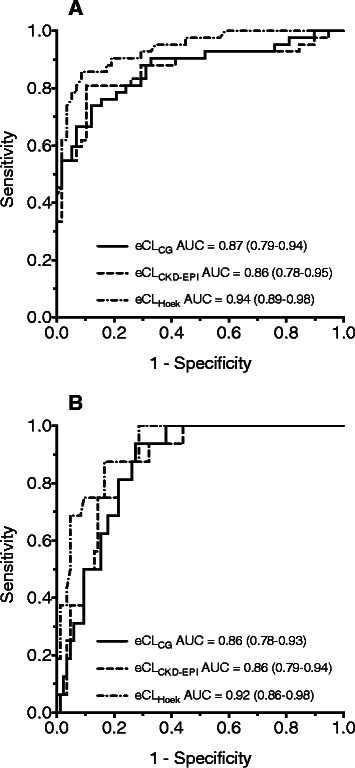
Receiver-operator characteristic curves for detection of reduced (**A**; CL_CR_ < 60 mL/min/1.73 m^2^) or augmented renal clearance (**B**; CL_CR_ > 130 mL/min/1.73 m^2^). AUC area under the curve (95%-confidence interval).

The sensitivity to detect low values of CL_CR_ (<60 mL/min/1.73 m^2^) was identically low for eCL_CG_ and eCL_CKD-EPI_, and significantly better (p < 0.01) for eCL_Hoek_. Specificity was >0.9 for all three estimates. Patients with augmented renal clearance (>130 mL/min/1.73 m^2^) were identified with the highest sensitivity by eCL_CG_, but specificity was significantly lower than that of the other two (p < 0.01; Table 2). The ROC curves both for low and high CL_CR_ showed a slightly higher discriminative power (in terms of AUC) for eCL_Hoek_ than for eCL_CG_ and eCL_CKD-EPI_ (Figure 3).

**Table 2:** **Sensitivity and specificity (95% confidence intervals) to detect reduced or augmented endogenous creatinine clearance**

	CL_CR_<60 mL/min/1.73 m^2^		CL_CR_>130 mL/min/1.73 m^2^	
	sensitivity	specificity	sensitivity	specificity
eCL_CG_	0.55 (0.39-0.70)	0.95 (0.86-0.99)	0.69 (0.41-0.89)**	0.81 (0.71-0.89)***
eCL_CKD-EPI_	0.55 (0.39-0.70)	0.97 (0.88-1.0)	0.25 (0.073-0.52)	0.96 (0.90-0.99)
eCL_Hoek_	0.83 (0.69-0.93)*	0.91 (0.81-0.97)	0.38 (0.15-0.65)	0.96 (0.90-0.99)

CL_CR_: measured endogenous creatinine clearance; eCL_CG_, eCL_CKD-EPI_, eCL_Hoek_: estimated clearance by the Cockcroft-Gault, CKD-EPI or Hoek formula.

*different from eCL_CG_ and eCL_CKD-EPI_ (p < 0.01).

**different from eCL_CKD-EPI_ (p < 0.05).

***different from eCL_CKD-EPI_ and eCL_Hoek_ (p < 0.01).

Since the variable duration of urine collection could have influenced the results, subsets were reanalysed which included only the patients of study 1 (n = 25), or all patients in which urine was collected for at least 12 hours (n = 66) or less than 8 hours (n = 31). The results did not show any relevant differences compared to the complete data set (Additional file 2).

## Discussion

With the present study, we provide data on the agreement of estimates of renal function based on plasma creatinine or cystatin C with measured endogenous creatinine clearance in ICU patients. In accordance with other studies [6,11], we observed extreme values of measured endogenous creatinine clearance in patients with plasma creatinine concentrations below the upper reference limit (≤0.8 mg/dL for women, ≤1.1 mg/dL for men), clearly illustrating the need for a more reliable quantification of renal function in this population.

Although the bias of all three estimates appeared acceptable, it is obvious that their precision is limited and may be completely misleading with regard to the actual renal function. Both creatinine based estimates tended to overestimate low or normal CL_CR_-values, which was also reflected by a low sensitivity to detect patients with reduced renal function. In this range, the cystatin C based Hoek formula was significantly more precise, and notably more sensitive with similar specificity. A plausible explanation for this is that cystatin C does not depend on muscle mass, unlike creatinine. All three formulae were derived from non-critically ill populations, and imply certain relationships between measures of body size (weight or BSA) and the production of cystatin C or creatinine. Loss of muscle mass due to immobilisation and catabolism is certainly a prominent feature in many critically ill patients, and necessarily affects the accuracy of creatinine based estimates. Cystatin C might therefore offer an advantage for assessing renal function both in clinical routine and research, when a timed urine collection or the clearance of an exogenous marker is not available. However, its exact significance must be further explored and defined, also to justify the much higher costs (in our institution ca. 10€ per determination compared with 0.50€ for creatinine). Although there are several reports on cystatin C-guided therapy with vancomycin or aminoglycosides [19-22], such informations are generally lacking for most drugs. Further open issues are the availability of standardised commercial assays, and confounding effects of various disease states. In contrast to initial reports, the production of cystatin C is not entirely constant either [12,23]. In the context of critical care, particularly inflammation, glucocorticoids and dysthyroid states could influence its levels, but also preexisting conditions such as obesity and smoking. Unfortunately, these confounding conditions were not assessed in our study, but given their rather generic nature it appears safe to assume that many patients with one or more of these conditions were represented in the study.

Baptista and colleagues made similar observations in 54 critically ill patients [9]. Although patients with plasma creatinine >1.3 mg/dL were excluded, 8 h-CL_CR_ ranged from 16.2 to 378.3 mL/min/1.73 m^2^, and the Cockcroft-Gault, the MDRD and the CKD-EPI formulae were unreliable (cystatin C was not determined). Villa and colleagues compared the reciprocals of plasma cystatin C and creatinine with 24 h-CL_CR_ in 50 critically ill patients at risk for developing acute renal failure, and reported a much stronger correlation for 1/cystatin C than for 1/creatinine [24]. Recently, Delanaye and colleagues reported data from a study in 47 critically ill patients, in which a true gold standard, iohexol clearance, was used [25]. Cystatin C was superior to creatinine to detect patients with GFR <60 mL/min, but patients with a plasma creatinine concentration >1.5 mg/dL were excluded from the study. In general, our results agree with these previous findings, but significantly extend the body of evidence in a large cohort (n = 100) of unselected (with regard to renal function) ICU patients.

Of concern is the lack of agreement of any of the three formulae with high CL_CR_-values, and their low sensitivity to detect patients with augmented renal clearance (CL_CR_ > 130 mL/min/1.73 m^2^), who are at risk for inappropriately low drug concentrations, e.g. of antibiotics. This again calls to mind that none of the formulae was designed for critically ill patients, but rather for detection and follow-up of chronic renal disease. When augmented renal clearance is suspected, it seems advisable to rely on the endogenous creatinine clearance rather than on estimates.

Several limitations must be mentioned. Our data were extracted as a secondary analysis from pharmacokinetic studies on renally eliminated antibiotic agents. Reliable quantification of renal function was therefore an important feature of the study designs, but some aspects were neglected, which would be necessary for a more thorough assessment of renal function parameters in critically ill patients. For one, renal function in critically ill patients is certainly dynamic. Glomerular filtration (as only the most important part of “renal function” in the context of drug dosing) can rapidly change due to e.g. hemodynamic alterations or systemic inflammation, two common events in ICU patients. Patients’ conditions were clinically judged to be “stable”, but more formal criteria (e.g. for vasopressor doses, fluid balance or urine output) would have been preferable. Second, the length of the urine collection periods were quite variable between patients, being only ~2 hours in some cases. Although such short periods have been reported to adequately substitute longer intervals (24 hours) [26], the results would be easier to interprete if a uniform protocol had been used for all patients, even if our sensitivity analysis did not show an influence of the duration of urine collection. Finally, endogenous creatinine clearance is also only an approximation of glomerular filtration. Besides free glomerular filtration, it is also subject to tubular secretion which becomes quantitatively more important when GFR decreases [27]. Tubular secretion of creatinine may be differentially affected by chronic or acute renal disease, and can be inhibited by a number of drugs (e.g. trimethoprim and cimetidine), which leads to a lower endogenous creatinine clearance despite constant GFR [27]. (In fact, blocking tubular secretion of creatinine by coadministration of cimetidine has been suggested as a mean to improve the accuracy of endogenous creatinine clearance measurements [28].) Cimetidine is not in use in the participating ICUs, and sulfamethoxazole-trimethoprim is administered only rarely (for prevention or treatment of *Pneumocystis jirovecii* infections); the prevalence of specific chronic disease (e.g. glomerulonephritis) was certainly minimal in our population of surgical ICU patients, whereas both degenerative loss of renal function and acute kidney injury probably accounted for the large part of patients with reduced CL_CR_. Unfortunately, however, a more detailed analysis is not possible because these informations were not systematically recorded. The use of a true gold standard, i.e. clearance of an exogenous marker such as inulin or iothalamate, would certainly have been desirable, but is unlikely to be done in clinical routine or on a large scale in clinical studies. As a compromise of validity and practicability, the use of endogenous creatinine clearance as reference, as done in the present study, seems therefore justified.

## Conclusions

We assessed the agreement of the Cockcroft-Gault formula, the CKD-EPI formula (both based on plasma creatinine) and the Hoek formula (based on cystatin C) with measured creatinine clearance in a comparatively large and unselected cohort of surgical ICU patients. In accordance with previous findings, extreme values of creatinine clearance were observed in patients with plasma creatinine concentrations below the upper reference limit. The Hoek formula was significantly more precise than the other two formulae. The value of cystatin C for clinical decision making and to guide drug dosing in ICU patients should be further defined (e.g. by inclusion in pharmacokinetic studies), before it may find a role in clinical routine. However, identification and quantification of augmented renal clearance without urine collection remains problematic.

## Additional files

Additional file 1:
**Anonymised clinical data and renal function estimates of the 100 study participants.**
Additional file 2:
**Separate analysis of subsets stratified for the duration of urine collection.**
