# Supplementary material for: Estimation of creatinine clearance using plasma creatinine or cystatin C: a secondary analysis of two pharmacokinetic studies in surgical ICU patients
Source: BMC Anesthesiol. 2015 Apr 28;15:62. doi: 10.1186/s12871-015-0043-7 (PMC4426534; doi:10.1186/s12871-015-0043-7)
Supplement: Additional file 2: — Separate analysis of subsets stratified for the duration of urine collection. [file 12871_2015_43_MOESM2_ESM.pdf]

Supplementary data to „Estimation of creatinine clearance using plasma creatinine or cystatin C: a secondary analysis of two pharmacokinetic studies in surgical ICU patients“. Separate analysis of subsets.

	Complete data set	Only study 1	Collection time≥12h	Collection time<8h
n	100	25	66	31
Agreement with CL_{CR}				
Bias eCL_{CG} (mL/h/1.73m^2)	+13.5	+16.4	+12.6	+14.2
Bias $eCL_{CKD\text{-}EPI}$ (mL/h/1.73m^2)	+7.59	+8.39	+4.81	+9.59
Bias eCL_{Hoek} (mL/h/1.73m^2)	-4.15	-3.92	-3.45	-6.98
Precision eCL_{CG} (mL/h/1.73m^2)	±18.5	±23.1	±18.6	±18.9
Precision $eCL_{CKD\text{-}EPI}$ (mL/h/1.73m^2)	±16.8	±20.1	±18.4	±15.6
Precision eCL_{Hoek} (mL/h/1.73m^2)	±12.9	±12.7	±12.1	±16.2
Detection of CL_{CR} <60 mL/min/1.73m^2				
Sensitivity/Specificity eCL_{CG}	0.55/0.95	0.88/1.0	0.62/0.93	0.54/1.0
Sensitivity/Specificity $eCL_{CKD\text{-}EPI}$	0.55/0.97	0.88/1.0	0.62/0.95	0.54/1.0
Sensitivity/Specificity eCL_{Hoek}	0.83/0.91	1.0/1.0	0.77/0.98	1.0/0.78
AUC ROC eCL_{CG}	0.87	1.0*	0.90	0.77
AUC ROC $eCL_{CKD\text{-}EPI}$	0.86	1.0*	0.90	0.78
AUC ROC eCL_{Hoek}	0.94	1.0	0.95	0.94
Detection of CL_{CR} >130 mL/min/1.73m^2				
Sensitivity/Specificity eCL_{CG}	0.69/0.81	0.60/0.70	0.67/0.76	0.75/0.89
Sensitivity/Specificity $eCL_{CKD\text{-}EPI}$	0.25/0.96	0.20/0.95	0.25/0.96	0.25/0.96
Sensitivity/Specificity eCL_{Hoek}	0.38/0.96	0.40/0.85	0.50/0.96	0.0/0.96
AUC ROC eCL_{CG}	0.86	0.78	0.83	0.91
AUC ROC $eCL_{CKD\text{-}EPI}$	0.86	0.86	0.87	0.83
AUC ROC eCL_{Hoek}	0.92	0.90	0.95	0.87

CL_{CR}: measured endogenous creatinine clearance; eCL_{CG}, $eCL_{CKD\text{-}EPI}$, eCL_{Hoek}: estimated clearance by the Cockcroft-Gault, CKD-EPI or Hoek formula; AUC ROC: area under the curve of the receiver operating characteristic.

*sensitivity/specificity would have been 1.0/1.0 for other cut-offs than 60 mL/h/1.73m^2, explaining the perfect AUC despite imperfect sensitivity/specificity.
